# PRM-D* Method for Mobile Robot Path Planning

**DOI:** 10.3390/s23073512

**Published:** 2023-03-27

**Authors:** Chunyang Liu, Saibao Xie, Xin Sui, Yan Huang, Xiqiang Ma, Nan Guo, Fang Yang

**Affiliations:** 1 School of Mechatronics Engineering, Henan University of Science and Technology, Luoyang 471003, China; chunyangliu@126.com (C.L.);; 2 Longmen Laboratory, Luoyang 471000, China; 3 Key Laboratory of Mechanical Design and Transmission System of Henan Province, Henan University of Science and Technology, Luoyang 471003, China

**Keywords:** probabilistic roadmap (PRM), D*, robotics, path planning

## Abstract

Various navigation tasks involving dynamic scenarios require mobile robots to meet the requirements of a high planning success rate, fast planning, dynamic obstacle avoidance, and shortest path. PRM (probabilistic roadmap method), as one of the classical path planning methods, is characterized by simple principles, probabilistic completeness, fast planning speed, and the formation of asymptotically optimal paths, but has poor performance in dynamic obstacle avoidance. In this study, we use the idea of hierarchical planning to improve the dynamic obstacle avoidance performance of PRM by introducing D* into the network construction and planning process of PRM. To demonstrate the feasibility of the proposed method, we conducted simulation experiments using the proposed PRM-D* (probabilistic roadmap method and D*) method for maps of different complexity and compared the results with those obtained by classical methods such as SPARS2 (improving sparse roadmap spanners). The experiments demonstrate that our method is non-optimal in terms of path length but second only to graph search methods; it outperforms other methods in static planning, with an average planning time of less than 1 s, and in terms of the dynamic planning speed, our method is two orders of magnitude faster than the SPARS2 method, with a single dynamic planning time of less than 0.02 s. Finally, we deployed the proposed PRM-D* algorithm on a real vehicle for experimental validation. The experimental results show that the proposed method was able to perform the navigation task in a real-world scenario.

## 1. Introduction

**Figure 1:**
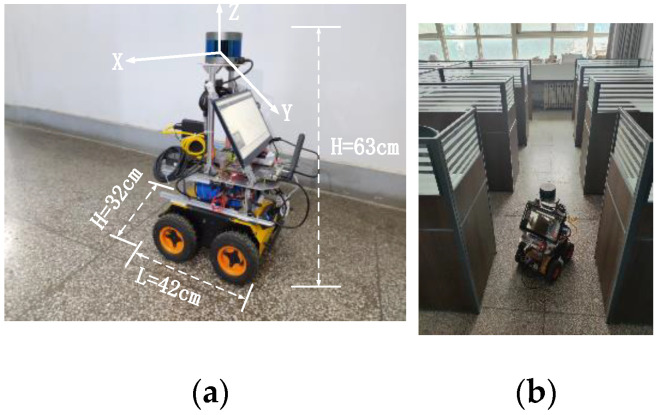
Indoor navigation task: (**a**) Four-wheel differential drive robot; (**b**) Office building as the deployment environment.

With the development of the times, the application of mobile robots is becoming more and more popular. Disaster relief robots, industrial transportation robots, inspection robots, etc., are moving deeper and deeper into our lives. Among many robot work scenarios, path planning work in large-range scenarios has been a difficult task. Because it needs to complete static planning to ensure the planning speed and path quality, it also needs to have the ability of dynamic planning. The wheeled robot we used is shown in Figure 1a, and its movement scenario is shown in Figure 1b. The goal was to determine end-to-end feasible paths and fast dynamic obstacle avoidance on a large-scale map. A path planning approach using deep learning seemed to be able to have both capabilities, but it required a long training time and is not suitable for deployment on an embedded platform. Therefore, we choose a traditional approach for the path planning task.

Common path planning methods include the genetic algorithm (GA), fuzzy logic algorithm (FA), D* algorithm [1], dynamic window approach (DWA), particle swarm optimization (PSO), ant colony optimization algorithm (ACO), and the probabilistic roadmap method (PRM) [2].

The initial versions of the aforementioned methods have various limitations in real-world applications; subsequently, researchers have improved these to overcome problems, such as increasing the number of variation operators to avoid the PSO algorithm falling into a local minimum [3] and increasing the number of crossover operators and fitness functions to avoid the fast convergence of the GA algorithm [4]. Furthermore, other methods have been utilized to address limitations. The adaptive fractional-order velocity algorithm was used to improve the PSO algorithm and minimize problems of falling into a local minimum and fast convergence [5]. The ACO algorithm has been improved using the sorting ant colony system to avoid falling into local traps [6]. The potential field method was employed to improve the planning success rate of PRM in narrow channels [7,8]. Weighted and automatic clustering methods have been used to reduce the planning time of D* [9,10]. Many researchers have introduced more environmental factors, such as the risk-based objective function [11] and road condition information [6], to improve the algorithm’s performance. In addition, a proportion of people chose to optimize parameters or paths. For example, the path was optimized by the Bessel curve to facilitate control, and the stability of the normalized robot was optimized by step size in the fuzzy control to reduce its motion error [12].

The above methods, which optimized the algorithms, are only applicable to specific environments and have significant limitations in a wide range of navigation tasks involving dynamic scenes, such as long operation times [6,11,13,14] and poor adaptation to dynamic environments [4,5,7,8].

PRM, as one of the classical path planning methods, constructs paths by randomly sampling in space, connecting sampling points, generating a network graph, and finally searching among discrete sampling points; it is characterized by a simple principle, probabilistic completeness, fast planning speed, and the asymptotic optimality of the formed path, but has poor performance in dynamic obstacle avoidance. When we used D* to plan a path in a wide range of scenes, the number of grids to be computed for graph searches increased dramatically due to the enlargement of the map area, which always led to excessive time overhead despite its dynamic planning speed being fast. Therefore, we propose a PRM-D* planning method for large-scale scenarios by combining PRM and D* algorithms, which introduces the D* algorithm in PRM to build a network that can reduce redundant sampling points and speed up the global planning speed of PRM; meanwhile, D* was used as a local planner in the execution phase to increase the dynamic performance of the algorithm, as a way to overcome the limitations encountered by these algorithms when they are employed individually.

The rest of this paper is organized as follows. In Section 2, we present related work. In Section 3, we discuss the PRM-D* algorithm and its three main improvement components, adding network edges, constructing local maps, and implementing dynamic obstacle avoidance. In Section 4, we show the differences between our method and other methods for maps of different complexity and experimentally validate the applicability of the proposed method in real-world scenarios. Section 5 concludes the whole paper. Finally, Section 6 describes the shortcomings of the method and future research directions.

## 2. Related Work

There are two different improvement ideas for solving path planning problems in complex scene environments using PRM methods.

One is to optimize the PRM algorithm itself. Bohlin et al. proposed the LazyPRM method, which turns PRM into a single-query fast planning method through the idea of delayed collision detection [15]. Karaman et al. proposed PRM* and LazyPRM* based on the improvement of PRM and LazyPRM, respectively, and eventually obtained the optimal path by increasing the number of contact points in the expansion gauge [16]. Dobson et al. increased the original single-layer network graph of PRM to two and proposed the SPARS algorithm, which accelerated the convergence speed by sparse and dense two-layer networks while possessing asymptotic optimality [17]. Thereafter, SPARS2 was proposed on this basis, and the interface calculation of the two-layer map was improved to further enhance the algorithm effect [18]. The above methods greatly improved the path search speed and shortened the path length of PRM. However, they did not solve the problems faced by PRMs in dynamic scenarios. For example, when the robot’s original route appeared to obscure obstacles, these methods require the re-planning of the overall path and could not meet the real-time nature of dynamic obstacle avoidance.

Another idea uses the idea of hierarchical programming to overcome the limitations of algorithms by combining different algorithms. The A* algorithm was used to plan the overall path, and PRM quadratic planning was used in the local scope to eliminate sharp turns in the path of A* planning [19]. However, in this approach, the global plan time of A* and the retraining time of PRM were high when dynamic obstacles conflicted with the map resolution. The PRM algorithm was used to estimate the general path, and the GA algorithm was used to calculate and improve branch point connections and optimize the wiring harness layout and reduce overall consumption [20]; however, this technique was applicable only for static environments. Rapidly exploring Random Trees (RRT) has been used to establish a global random search tree, and the Reinforcement Learning (RL) algorithm has been employed as a local planner and controller for optimizing the extension of RRT [21]. However, the training time is too long, the scene portability is poor, and the actual planning is time-consuming. Subsequently, a method based on the combination of PRM and RL was proposed [22]; PRM was used to preliminarily plan the path, and RL was used to act as a local agent. RL was used to train each local area in detail and then splice it. Compared with the previous method [21], this method was faster and could realize fine-obstacle avoidance movements. However, if the number of obstacles suddenly increased, there was a high risk that this method would not work. To address the problem of traditional deep learning algorithms requiring long development time, Gao Jun li et al. proposed a phased cultivation method PRM- TD3 to shorten the development time, which consists of PRM undergoing global planning TD3 and conducting local training for better flexibility in single-step time but performing poorly in overall path time [23]. In a multi-robot cooperative task, Semiz, Fatih et al. performed initial planning based on the conflict search and used D* for the underlying planning of individual robots to improve resilience in dynamic scenarios [24]. D* has been used to determine the path cost node, and PSO has been used to optimize the control execution trajectory at the execution level [25]. The curve is better in a dynamic environment, but D* consumes more time in a large range of scenes. As for the slow search time of D* in large-scale scenes, Hu Huang et al. reduced the path planning time by 1/3 using the D* method with improved heuristic functions in vector maps [26], but the planning time was still too long compared to methods such as PRM.

The aforementioned improved algorithm proves the effectiveness of PRM in global planning and the flexibility of the D* algorithm in dynamic environments. Therefore, in this study, we used PRM for initial planning and D* for local planning. The success rate of the PRM path planning was improved, the number of PRM path queries was reduced, the planning speed improved, and the introduced D* algorithm optimized the path length of PRM planning to a certain extent while making PRM have a strong dynamic planning capability.

## 3. Method

Improvements to PRM can be divided into two major parts, namely, improvements in building networks and improvements in dynamic planning capabilities. The former speeds up the planning success rate and planning speed of the PRM, and the latter gives PRM the ability of dynamic obstacle avoidance. The building network phase was conducted by making changes to the pathfinding method among PRM nodes in order to reduce the number of network nodes and speed up the pathfinding. Dynamic planning can be achieved by constructing a local map dependent on the main path, and the dynamic planning capability of the algorithm was enhanced by introducing the D* algorithm.

### 3.1. PRM Construction

**Figure 2:**
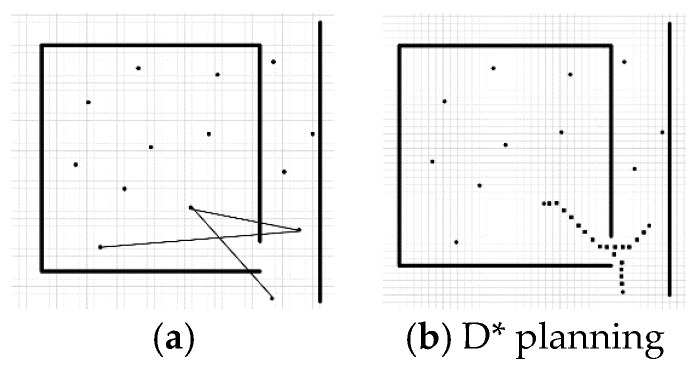
Different ways to build edges: (**a**) Line connection (**b**) D* connection.

In the classical PRM method, the barrier-free path between nodes is utilized to connect two points in a straight line and judge whether they pass through the obstacle. This method yields a fast calculation speed; however, in practical applications, it often leads to the failure of connectivity in narrow positions. As shown in Figure 2a, it can be assumed that the interior of the box is the interior and the space between the lines is the corridor; the indoor and corridor are connected through doors, and sampling points on both sides are easily blocked. Increasing the sampling points solves this problem but creates a large number of redundant points, which increases the duration of the path-planning phase.

Therefore, we used the D* algorithm to perform secondary retrieval when the road map node failed to pass a straight-line edge construction so that it could bypass the simple occlusion in the neighborhood range, as shown in Figure 2b.

Algorithm 1 describes how PRM-D* adds edges to the PRM. In classical PRM, the connection cost of a straight-line edge was determined by calculating the Euclidean distance between two points. To match the weight of the D* planned path and the original PRM edge, the distance of the points with the same x- or y-axis coordinates in the path array was denoted as 1, and the distance of the remaining adjacent points was denoted as 1.4. For example, if you move from the grid (0, 0) to (2, 1), its walking path is (1, 0), (2, 1), then the connection cost of this path is (1 + 1.4) * grid resolution. Finally, the weight of the path was obtained by summing up the distance between all points and multiplying the obtained value with the grid resolution.
**Algorithm 1:** Addition of edges in PRM-D* 1. weight ← 0 2. for i = 1 … N do 3. for j = i +1 … N do 4.  if F(si, gj) then 5.   weight ← O(si, gj) 6.   if weight <DIS then 7.    add_ edge ← [si, gj, weight] 8.  else: 9.   try: 10.    path ← D-S(si, gj) 11.    weight ← W(path) 12.    if weight <DIS then 13.     add_ edge ← [si, gj, weight] 14.   except: 15.     pass 16. return add_ edge

When judging whether the sampling points are connected, s, g is the current calculation of two sampling points; point N is the total number of sampling points, O is the path cost between two sampling points when they can be connected in a straight line, W is the path cost between two sampling points when they cannot be connected in a straight line but can be connected using D*, DIS determines whether the cost is too high after D* connection; in a complex environment, the actual connection path of points close to the spatial distance may be very long. F determines whether the two sampling points can be connected in a straight line.

**Figure 3:**
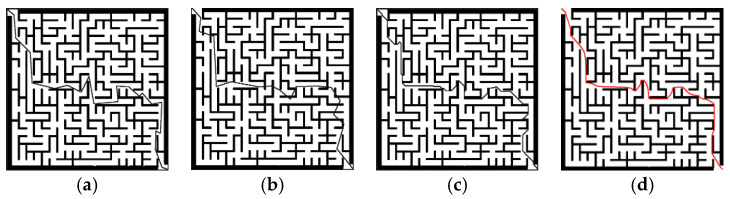
PRM- and PRM-D*-generated paths in the complex map: (**a**) is the PRM path, (**b**) is the modified PRM path, (**c**) is the path after introducing D*, and (**d**) is the final path.

The planning results are shown in Figure 3. The improved PRM first generates the path after linear connection, then invokes the local planning results of D* to generate Figure 3c. However, the path at this point contains many corners, which are difficult to use for robot walking control. B spline is a common curve interpolation optimization method, and it can also enhance local modification through the control points, so we choose to use B spline to optimize the original path.

### 3.2. Local Map Construction

**Figure 4:**
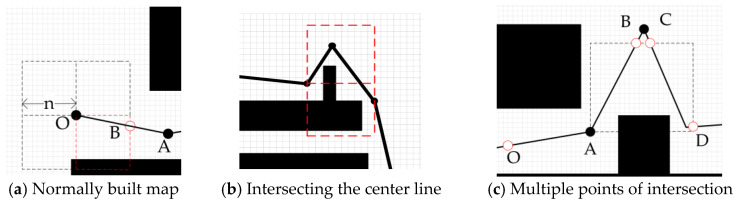
Different local map builds. The selected local map in (**a**) is the red dotted box selection range, and (**b**,**c**) omits the unselected map.

Algorithm 2 describes the process of local map construction. The construction of local map boundaries under normal conditions is illustrated in Figure 4a. The local map boundary range n is usually around 50 raster distances; if n is too small, it will affect dynamic obstacle avoidance, and a too large n will lead to an increase in D* local search time. The black squares in the figure represent obstacles, and points O and A are path nodes generated by the PRM algorithm. The robot moves from point O to point A, the coordinates of point O are (x, y), and n is the size of the local map. With x+n as fixed X-axis coordinate parameters and y+n to y-n as Y-axis coordinate parameters, the set of the right bounding box is formed, and similarly, the left bounding box and upper and lower bounding boxes can be generated. The intersection point B of the border and PRM path is the target point of the local map. The cross centerline is constructed similarly to the border, except that the (x, y) coordinates are no longer offset by a distance n. If the center line of the cross has no intersection with the PRM path, the final path is the part selected by the red dotted box. In the case of an intersection between the path and the center line of the cross, as shown in Figure 4b, the local map with an intersection at the cross line is retained.
**Algorithm 2:** Local map construction1. N_L_
← S, N_0_
← S, N_1_
← S, I_n_
← 0 2. if N_0_ != G then 3. L_E_
← L_ES_ (N_0_,n) 4. I_n_,P ←
**Intersection** (L_E_, Pp) 5. if I_n_ == 1 then 6.  N_1_
← P 7.  P_E_ ← P_ES_(N_0_,n) 8.  i_n_,p ←
**Intersection** (P_E_,Pp) 9.  if i_n_ is not empty then 10.   Local_ edge ← // Preserve the boundary of the half region where p is located 11.  else 12.   Local_edge ← // Preserve the boundaries of the quarter area where the N_1_ is located 13.  Pp ← // Pp removes the pathpoint before the N_0_ coordinate 14. elif I_n_ > 1 then 15.  if N_L_
== N_0_ then // initial position 16.  $\dot{O_{n}}$
←
**vector** (N_0_, P) 17.  //Calculate the angle α between $\dot{O_{n}}$ and $\dot{\theta }$
18.   N_1_ ← **min**(P,α) // The vector with the smallest Angle 19.  //Repeat 7 ~ 13 20.  else 21.   $\dot{\theta }\;$← **vector** (N_L_, N_0_) 22.  //Repeat 16~19 23. else 24.  N_1_ ← G 25. //Repeat 7~13 26. local_map ← **local**(local_edge, glogal_map) 27. return local_map 28. else 29. return End of the navigation

Figure 4a,b shows the situation corresponding to a single intersection point between the border and PRM path. In the case of multiple intersections, vectors must be constructed to help select local target points. As shown in Figure 4c, vector *OA* is formed with *O* as the starting point, and *AB*, *AC*, and *AD* constitute three vectors. The included angle between vectors *OA* and *AB* can be calculated as follows:

(1) $$\dot{OA}=\left(x_{1},y_{1}\right)=\left(x_{a}-x_{o}\;,\;y_{a}-y_{o}\;\right)$$

(2) $$\dot{AB}=\left(x_{2},y_{2}\right)=\left(x_{b}-x_{a}\;,\;y_{b}-y_{a}\right)$$

(3) $$\cos\left(\dot{OA},\;\dot{AB}\right)=\left[x_{1}y_{1}+x_{2}y_{2}\right]/\left[\sqrt{\left(x_{1}^{2}+y_{1}^{2}\right)}\times \sqrt{\left(x_{2}^{2}+y_{2}^{2}\right)}\right].$$

(4) $$\theta =arccos\left(\cos\left(\dot{OA},\;\dot{AB}\right)\right)$$

The point with the lowest angle is selected as the next node.

In this logical segment, S, G are the starting and target points under the global map. The map planned by the PRM is formed by connecting several nodes, and the robot builds a new local map when it walks to a node. At this point, the node where the robot is currently located is denoted as N_0_, the node it has just walked past is denoted as N_L_, and the node it is going to is denoted as N_1_. When building the local map, we first built a large bounding box around the node, i.e., L_E_, and the function to build L_E_ was L_ES_. To reduce the planning time of D* in the local map, we chose to use two vertical intersecting medians to divide L_E_ into four. A large bounding box, L_E_, was constructed around the node, and the function to construct L_E_ was L_ES_. In order to reduce the planning time for D* in the local map, we needed to reduce the local map area as much as possible, so we chose to divide the L_E_ into four parts using two vertically intersecting medians, this median boundary line is denoted by P_E_, and P_ES_ is the function for constructing P_E_. Pp is the global path.

### 3.3. Dynamic Obstacle Avoidance

**Figure 5:**
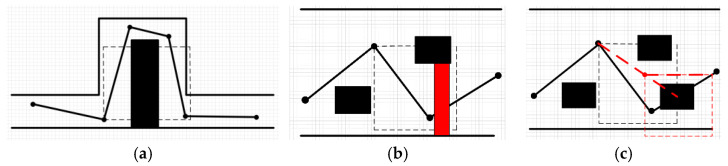
Different special cases: (**a**) Obstacles beyond the local map, (**b**) A sudden obstacle obscuring all feasible paths in the local map. (**c**) A target point obscured by a dynamic obstacle.

For most scenes, the local map constructed using the aforementioned algorithms could meet the planning needs. However, as shown in Figure 5a, for the global planning, the path was connected, but for the intercepted local map, there was no feasible path between the starting point and the target point. Or as shown in Figure 5b, the sudden obstacle blocks all provide feasible routes in the local map but do not affect the selection of nodes. At this point, the construction of the local map was abandoned, and the robot location and N1 were selected as the starting point and end point, respectively, to construct the global map for D* planning. This method works well for these special scenarios; however, there is a slight increase in planning time when boundary constraints are lost. If obstacles block the target node, as shown in Figure 5c, the point where the current robot is located, that is, N0, is used to reconstruct the local map and plan the local path.

The specific process is shown in Algorithm 3.
**Algorithm 3:** Dynamic obstacle avoidancelocal_path ← []if move_obs is empty then try:  local_path ← D_S(s, g, local_map)// D_S:D* agent’s policy  return local_path except:  local_path←D_S(s, g, glogal_map)  return local_pathelse local_map ← **local**(local_map, move_obs) try:  Local_path ← D_S_updata(s, g, local_map) except:  new_glogal_map ← glogal_map +move_obs  if g∈move_obs then   return []//Returns a null value, restart the map building process  else   try:    local_path ← D_S_updata(s, g, local_map)    return local_path   except:    local_path ← D_S_updata(s, g, new_glogal_map)    return local_path

## 4. Experiment and Analysis

### 4.1. Basis of Experiment

**Figure 6:**
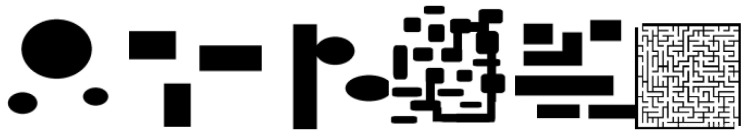
Raster maps used in the experiment arranged in the order of increased difficulty.

(1) Robot setup: A differential drive was used for robot kinematic models. The robot’s movement was controlled at 20 Hz, and the obstacles observed by the depth camera were projected onto the plane map through coordinate system transformation and were represented as a whole composed of squares 0.1 m. The detection frequency of the camera was set at 30 frames per second.

(2) Map selection: Figure 6 shows the grid maps selected for simulation according to different complexities. The resolution of the grid maps was set as 500 × 500, and the size of one grid was set as the size of the robot.

(3) Roadmap Construction: For simplicity, we used uniform random sampling to construct the roadmaps. The construction range N of the local map was usually 50 times the size of the robot. Therefore, in point-to-point PRM network construction, the Euclid distance between a single point and a point generally does not exceed the distance N unless otherwise stated to prevent errors in the navigation stage and reduce the time cost of edge construction.

(4) Parameter selection: PRM-D* can be compared with classical PRM, D*, GA, fuzzy methods, and the SPARS2 method from the OMPL library. In the edge building phase of PRM, a straight-line connection was used, and the neighborhood distance was chosen to be 100 to meet the construction requirements of a simple map. the maximum number of iterations of GA was chosen to be 50 to prevent the operation time from being too long. Since the parameters of the fuzzy method needed to be adjusted in maps of different complexity, Fuzzy manually adjusted the parameters according to different maps.

### 4.2. Performance Comparison before and after PRM Improvement

According to the principle of improved edge addition in PRM, when the local map is simple, the occlusion is small, and there is little difference in the algorithm performance before and after improvement. Therefore, only the raster map with the highest complexity was selected here to discuss the performance comparison of the PRM algorithm before and after improvement.

**Figure 7:**
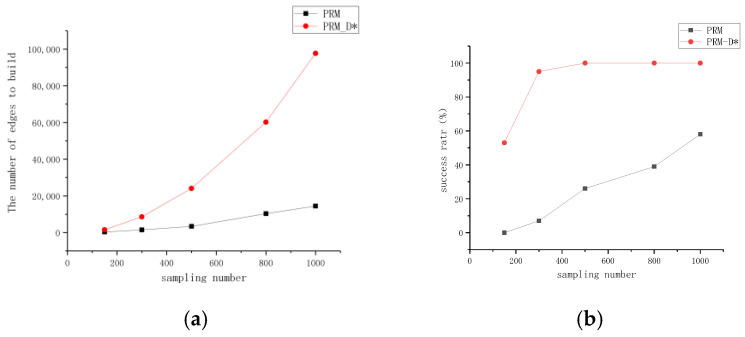

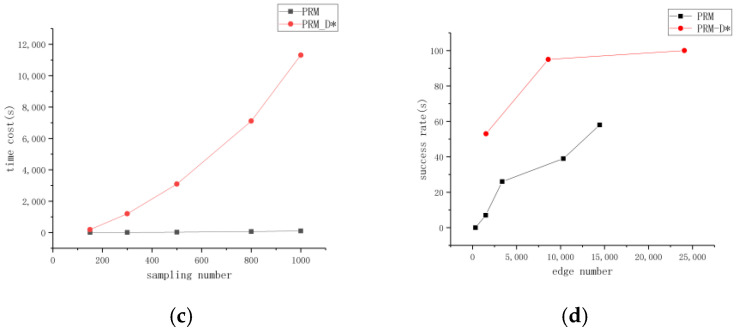
Performance comparison of PRM and PRM-D* in network construction. (**a**) Number of sampling points versus number of edges required. (**b**) Relationship between number of sampling points and success rate. (**c**) The number of sampling points versus the time required for network construction. (**d**) Relationship between number of edges and success rate.

**Figure 8:**
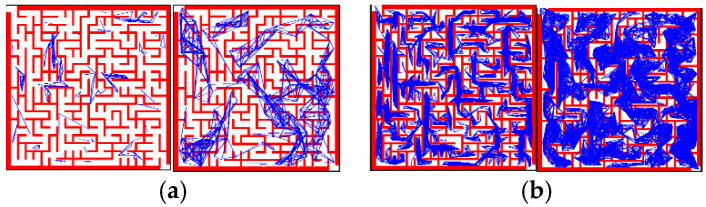
Network construction under different numbers of sampling points: (**a**) Comparison of PRM and PRM-D* composition for 150 sampling points; (**b**) Comparison of PRM and PRM-D* composition for 1000 sampling points.

**Figure 9:**
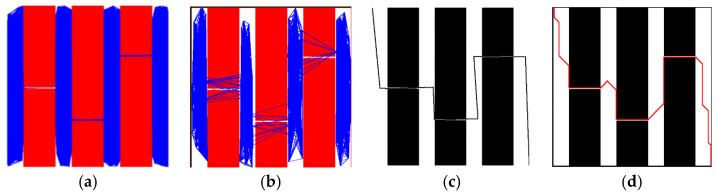
Slit map planning results: (**a**) Classical PRM network at 800 sampling points; (**b**) PRM-D* network at 150 sampling points; (**c**,**d**) are the final results for PRM and PRM-D* after planning, respectively.

To verify the performance comparison of PRM before and after improvement, we selected different numbers of the sampling points in the same map and conducted experiments and analyses in terms of three aspects: the number of edges built, the success rate of the path planning, and time consumption. The experimental data included the average results of each group of experiments performed 100 times. As can be seen from Figure 7a–c, with the increase in the number of sampling points, both PRM and PRM-D* exhibited an overall upward trend in terms of all three aspects. However, under the same number of sampling points, the improved PRM-D* exhibited far more edges and a higher success rate than the PRM method. When the number of sampling points was 300, the difference was the largest, and the difference in the success rate was 88%. The time cost of PRM-D* in network construction, however, was much higher compared to that before the improvement. This is because we did not limit the upper limit of the paths between the sampled points searched by D* when we performed the validation in order to explore gaps in the network construction. This can be seen in Figure 8a, where there are long network edges in the right-hand image. However, in the slit experiment, as shown in Figure 9b, after increasing the upper limit of the search, there was only a limited number of connected lines, at which time we found that after setting the search range of D* to be equal to the neighborhood distance of PRM, the network construction time took usually two to three times longer than that of the traditional PRM method, which varied according to the complexity of the map.

An analysis of the success rate data of PRM-D* revealed that although an overall upward trend was observed, when beyond a certain number of sampling points, increasing the number of sampling points did not result in more improvements. For 300 sampling points, the success rate was 95%, and for 500 sampling points, the success rate was 100%. This indicates that for a given complexity, there was an upper limit for the number of sampling points, and the number of sampling points could be reasonably selected according to the complexity of the map.

The comparison of the number of edges constructed by PRM and PRM-D* for different numbers of sampling points with the success rate in Figure 7d revealed that the number of edges for PRM at 1000 sampling points was more than twice the number of edges for PRM-D* at 500 sampling points; however, the success rate of PRM was only 0.6 times that of PRM-D*, and combined with Figure 8, it could be seen that the traditional PRM method had fewer effective edges at corner locations. After the improvement, the number of corner edges increased.

**Figure 10:**
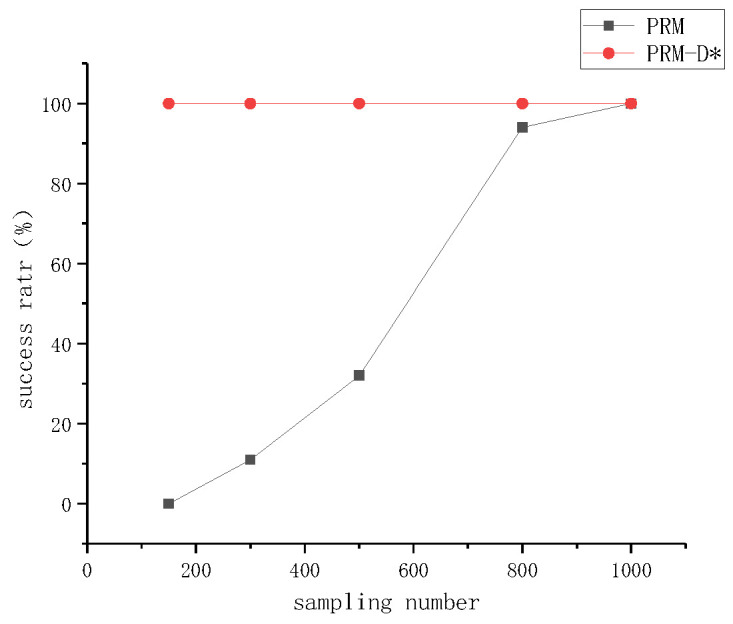
Comparison of the success rate of PRM and PRM-D* for each sampling point in the slit map.

The difference between PRM-D* and PRM could be more easily observed in maps with slits. The classical PRM algorithm often requires more sampling points to obtain the required success rate when facing a slit map. We started the experiment from 150 sampling points and repeated the network construction 20 times for each additional 50 sampling points to verify the probability of successful path planning. The conventional PRM method could guarantee a planning success rate of 90% after only 800 sampling points. By contrast, the improved method required only 150 sampling points to obtain better results, as shown in Figure 10. The reason for this can be easily derived from Figure 9, which shows that the classical method had some network edges near the slit, even at 800 sampling points. The improved PRM-D* algorithm performed well in dealing with the slit scenario.

### 4.3. Influence of Sampling Density on Planning Speed and Path Quality

From the results in the previous section, we know that in a complex map such as Figure 6, 150 sampling points had a planning success rate of 50%, 500 sampling points had a success rate of just reaching completion success, and 1000 sampling points created a large amount of redundancy. Therefore, to verify the effect of different sampling points on the overall planning speed and planning quality, we selected 150, 500, and 1000 sampling points and analyzed the secondary planning speed and path length when the number of sampling points changed on raster maps with different complexities.

**Figure 11:**
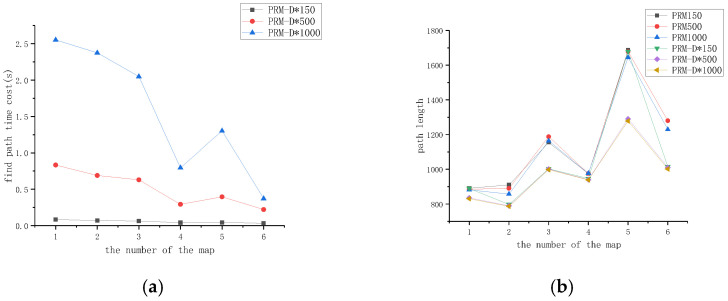

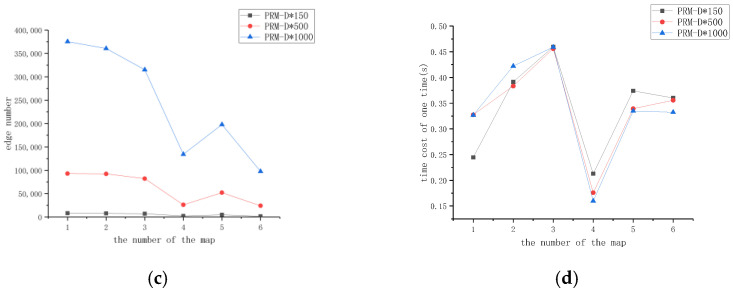
Performance comparison of PRM and PRM-D* with different parameters in different maps during the path planning phase. (**a**) Path planning duration under different maps; (**b**) Path length in different maps; (**c**) Relationship between the number of sampled points and edges in maps of different complexity; (**d**) Time required to construct a local map in a single pass in different maps.

From Figure 11a, it can be seen that the path-planning time consumed by the same-map PRM increased with the number of sampling points, and the path-planning time of different-map PRM decreased with an increase in map complexity. From Figure 11c, the reason can be noted that the higher the complexity of the map, the smaller the number of established edges. Therefore, in practical applications, the number of sampling points can be adjusted according to the complexity of the environment to achieve a balance between the success rate and time overhead.

Figure 11b depicts variations in the path length for PRM-D* and PRM for different numbers of sampling points. Overall, the path length of PRM-D* was smaller than the path generated by PRM regardless of the number of sampling points and the complexity of the maps, which proves that the original PRM path was optimized after incorporating the D* algorithm. Looking at each map alone, the increase in the number of sampling points had a greater effect on the PRM path length, while the impact on PRM-D* was smaller, which proved that the optimization of D* based on the original path of PRM was sufficient to compensate for the optimization results of the PRM path, which was caused by an increase in the number of sampling points by a certain amount.

The effect of increasing the number of sampling points on the single planning time is shown in Figure 11d; the overall trend of the local planning speed was the same as the map changes, which indicates that the effect of map complexity on the planning time was much greater than that of the number of sampling points.

### 4.4. Comparison of PRM-D* with Other Methods

In Section 4.3, we discuss the success rate of PRM-D*. Here, we used different methods on all the maps to compare and validate the performance of PRM-D* in terms of planning time and path length. From the analysis of the results in the previous sections, we selected 500 sampling points, which could ensure sufficient planning success with a low time overhead as the base parameters for PRM-D*. In the comparison process, the path length and planning time of PRM-D* are the sum of all local planning results. The path length of the non-raster method was calculated by fitting it with the centroid spacing of the raster through which it passed.

**Figure 12:**
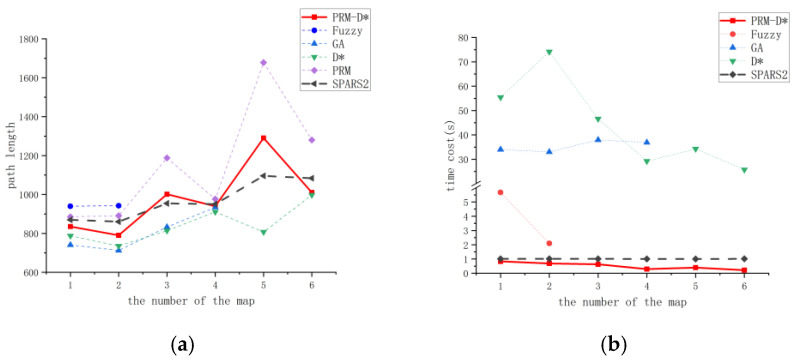
Time and path overhead of each method in different maps. (**a**) Length of paths generated by different algorithms on different maps. (**b**) Time required for different algorithms to plan paths on different maps.

Figure 12a,b shows the length and time consumption of the paths planned by each method in different maps. In terms of the path length, the graph search-based D* algorithm was optimal among the methods, but the search time consumption had too much overhead compared with the PRM-related improvement algorithm. Comparing this method with the SPARS2 method, the different maps both had advantages and disadvantages in terms of path length, and the overall performance was approximately the same considering the randomness of PRM-generated path nodes. In terms of planning time overhead, the proposed method was slightly faster than the SPARS2 method when the sampling points were taken to be 500. Experimentally, this method proved to be very competitive in static pathfinding.

### 4.5. Dynamic Obstacle Avoidance

To verify the dynamic obstacle avoidance performance of PRM-D*, we calculated the time required for PRM-D* to replan in the case of the sudden appearance of obstacle points by manually adding points and, thus, changing the original path. When adding different obstacles, it is difficult to guarantee that both methods have the same path change length when avoiding obstacles; therefore, an approximate interval was used to measure the level of replanning. The final time was chosen as the average of 20 sets of data.

**Figure 13:**
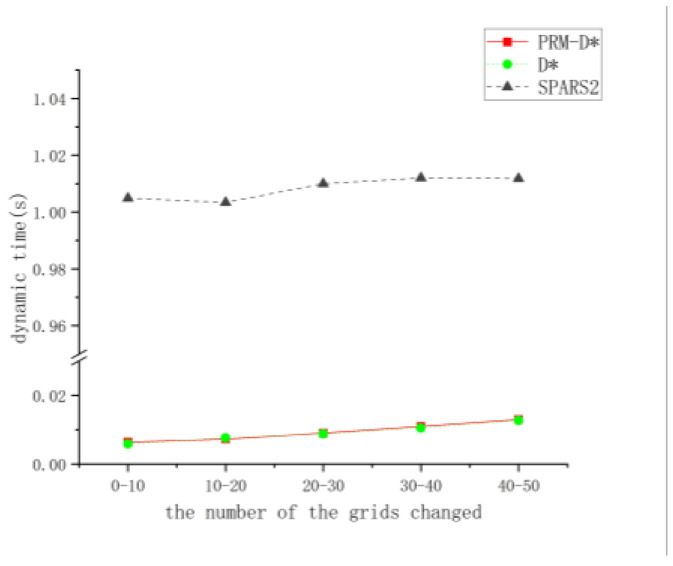
Dynamic planning time comparison.

Figure 13 shows the replanning time required for different levels of path changes when different obstacles were encountered. The comparison of the replanning times for different intervals shows that the proposed PRM-D* method did not differ significantly from D* when faced with similar environmental changes; the time spent by both is almost linear when replanning for grid number changes, while the SPARS2 method still required a new planning effort when encountering map changes, it thus had poor dynamic obstacle avoidance, compared to which our method was nearly two orders of magnitude faster in dynamic planning; thus, it can be demonstrated that PRM-D* yields a good dynamic performance under usual circumstances.

### 4.6. Autonomous Movement and Dynamic Obstacle Avoidance

**Figure 14:**
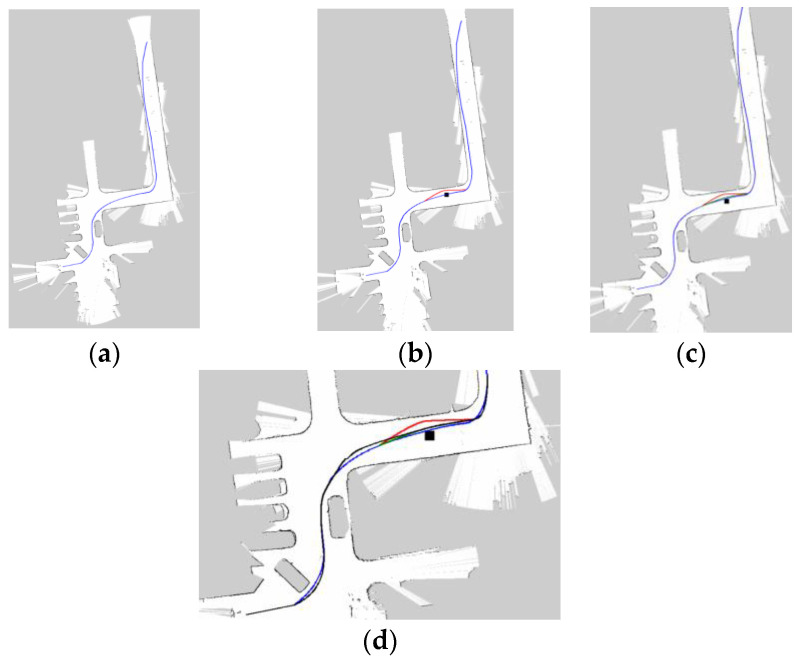
Scenario map and planning in real-world experiments. (**a**) Initial planning of the path. (**b**) Change of route when an obstacle appears. (**c**) The route changes again after the obstacle moves. (**d**) The planned path and the actual path traveled by the robot.

In the previous subsections, we discussed the performance of PRM-D* in raster maps. In this section, we verify the effectiveness of our method for planning a real scene. The experiments were carried out on a section of street in a campus with an overall size of 96 m × 24 m. After determining the start and target points, the initial planning results are shown in Figure 14a. The robot acquires the 3D spatial information of the target for target recognition through a depth camera mounted in the center of the robot and performs a coordinate system transformation to project the obstacle into a 2D SLAM map of the obstacle. We arranged for a human to push the obstacle so that it could move laterally during the robot’s walk, and when the robot detected the obstacle, its change trajectory is shown as the red trajectory in Figure 14b. As the obstacle moved rapidly, the robot again planned a path, as shown in the green trajectory in Figure 14c. The robot’s walking trajectory was generated from the LiDAR data, and its final path trajectory is shown as the black trajectory in Figure 14d. The experiments demonstrate that our method can be used in real-life scenarios.

## 5. Conclusions

The PRM method has become one of the classical path planning methods because of its simple and easy-to-understand principle and its probabilistically complete and asymptotically optimal characteristics, but the lack of a dynamic obstacle avoidance capability is always a pressing problem. In this study, a hierarchical planning method was developed by combining the PRM and D* algorithm. Through experiments and comparisons, the following conclusions were drawn: (1) The improvement of the add-edge algorithm enables the PRM algorithm to achieve a higher success rate with fewer sampling points, and the reduction in the number of sampling points greatly reduces the time cost of the path planning process. The global planning speed is slightly better than the spars2 method when the effective sampling points are selected at 500. (2) PRM-D* greatly improves the dynamic obstacle avoidance capability while ensuring fast global planning and increases the dynamic planning speed by two orders of magnitude compared with spars2; (3) Although the path generated using PRM-D* is not optimal, the global path length was smaller than that achieved using the original PRM, and was close to that generated by spars2, which was competitive; (4) Experimental. The results show that the robot can achieve navigation and dynamic obstacle avoidance in a wide range of maps. In summary, this study uses PRM as the basis and introduces D* to complete the network construction, giving a new way to accelerate the planning by constructing more effective edges, reducing the need for sampling points, and speeding up the path generation in the query phase; in addition, using D* as a local planner with the idea of hierarchical planning can achieve fast dynamic planning in the local range, making the method applicable to dynamic scenes.

## 6. Discussion

In this paper, we experimentally validated the feasibility of our algorithm and the competitive planning performance it exhibits in static and dynamic scenarios through different perspectives. However, there are still shortcomings compared with other methods, such as the optimality of static paths. The spars2 method of making paths asymptotically optimal by generating additional nodes near existing path nodes in an iterative way is a good approach, and better paths can have less motion overhead at the beginning of planning or other scenarios where planning efficiency is less demanding. In future work, we will investigate the optimality of the paths.

In addition, the method in this paper does not consider the difference in energy overhead in terms of straight ahead, turning, and in situ steering in conjunction with the motion process during the study and only improves the algorithm in terms of path length and planning speed. Combining kinematic principles to generate paths that are more consistent with the motion process will also be the focus of future research.

## Data Availability

Not applicable.

## References

[1] Stentz A. (1997). Optimal and Efficient Path Planning for Partially Known Environments. Intelligent Unmanned Ground Vehicles.

[2] Kavraki L., Svestka P., Latombe J.-C., Overmars M. (1996). Probabilistic roadmaps for path planning in high-dimensional configuration spaces. IEEE Trans. Robot. Autom..

[3] Qin Y.Q., Sun D.B., Li N., Cen Y.G. Path planning for mobile robot using the particle swarm optimization with mutation operator. Proceedings of the 2004 International Conference on Machine Learning and Cybernetics (IEEE Cat. No. 04EX826).

[4] Lamini C., Benhlima S., Elbekri A. (2018). Genetic Algorithm Based Approach for Autonomous Mobile Robot Path Planning. Procedia Comput. Sci..

[5] Song B., Wang Z., Zou L. (2020). An improved PSO algorithm for smooth path planning of mobile robots using continuous high-degree Bezier curve. Appl. Soft Comput..

[6] Song Q., Zhao Q., Wang S., Liu Q., Chen X. (2020). Dynamic Path Planning for Unmanned Vehicles Based on Fuzzy Logic and Improved Ant Colony Optimization. IEEE Access.

[7] Chen J., Zhou Y., Gong J., Deng Y. An improved probabilistic roadmap algorithm with potential field function for path planning of quadrotor. Proceedings of the 2019 Chinese Control Conference (CCC).

[8] Ravankar A.A., Ravankar A., Emaru T., Kobayashi Y. (2020). HPPRM: Hybrid Potential Based Probabilistic Roadmap Algorithm for Improved Dynamic Path Planning of Mobile Robots. IEEE Access.

[9] Liu X., Deng R., Wang J., Wang X. (2014). COStar: A D-star Lite-based dynamic search algorithm for codon optimization. J. Theor. Biol..

[10] Heo S.-N., Chen J., Liao Y.-C., Lee H.-H. (2022). Auto-splitting D* lite path planning for large disaster area. Intell. Serv. Robot..

[11] Al Hilli A., Al-Ibadi M., Alfadhel A.M., Abdulshaheed S.H., Hadi A.H. (2021). Optimal path finding in stochastic quasi-dynamic environments using particle swarm optimization. Expert Syst. Appl..

[12] Guo J., Li C., Guo S. (2019). A Novel Step Optimal Path Planning Algorithm for the Spherical Mobile Robot Based on Fuzzy Control. IEEE Access.

[13] Zhong X., Tian J., Hu H., Peng X. (2020). Hybrid Path Planning Based on Safe A* Algorithm and Adaptive Window Approach for Mobile Robot in Large-Scale Dynamic Environment. J. Intell. Robot. Syst..

[14] Wilt C., Ruml W. When does weighted A* fail?. Proceedings of the International Symposium on Combinatorial Search.

[15] Bohlin R., Kavraki L.E. Path Planning Using Lazy PRM. Proceedings of the IEEE International Conference on Robotics and Automation.

[16] Karaman S., Frazzoli E. (2011). Sampling-based Algorithms for Optimal Motion Planning. Int. J. Robot. Res..

[17] Krontiis A., Dobson A., Bekris K. Sparse Roadmap Spanners. Proceedings of the Workshop on the Algorithmic Foundations of Robotics (WAFR).

[18] Dobson A., Bekris K. Improving Sparse Roadmap Spanners. Proceedings of the 2013 IEEE International Conference on Robotics and Automation (ICRA).

[19] Chowdhury M.I., Schwartz D.G. The PRM-A* path planning algorithm for UUVs: An application to Navy mission planning. Proceedings of the Global Oceans 2020: Singapore–US Gulf Coast.

[20] Zhao Y., Liu J., Ma J., Wu L. (2021). Multi-branch cable harness layout design based on genetic algorithm with probabilistic roadmap method. Chin. J. Mech. Eng..

[21] Chiang H.-T.L., Hsu J., Fiser M., Tapia L., Faust A. (2019). RL-RRT: Kinodynamic Motion Planning via Learning Reachability Estimators From RL Policies. IEEE Robot. Autom. Lett..

[22] Francis A., Faust A., Chiang H.T.L., Hsu J., Kew J.C., Fiser M., Lee T.W.E. (2020). Long-range indoor navigation with prm-rl. IEEE Trans. Robot..

[23] Gao J., Ye W., Guo J., Li Z. (2020). Deep Reinforcement Learning for Indoor Mobile Robot Path Planning. Sensors.

[24] Semiz F., Polat F. (2020). Incremental multi-agent path finding. Future Gener. Comput. Syst..

[25] Sadiq A.T., Hasan A.H. Robot path planning based on PSO and D* algorithmsin dynamic environment. Proceedings of the 2017 International Conference on Current Research in Computer Science and Information Technology (ICCIT).

[26] Huang H., Huang P., Zhong S., Long T., Wang S., Qiang E., Zhong Y., He L. Dynamic Path Planning Based on Improved D* Algorithms of Gaode Map. Proceedings of the 2019 IEEE 3rd Information Technology, Networking, Electronic and Automation Control Conference (ITNEC).

